# Applying Lean Healthcare in the hospitalization and patient discharge process: an integrative review

**DOI:** 10.1590/0034-7167-2022-0751

**Published:** 2023-11-10

**Authors:** Livia Barrionuevo El Hetti Fuentes, Lucas Gardim, Thaís Oliveira da Silva, André Almeida de Moura, Andrea Bernardes

**Affiliations:** I Universidade de São Paulo. Ribeirão Preto, São Paulo, Brazil; II Universidade Federal de Minas Gerais. Belo Horizonte, Minas Gerais, Brazil

**Keywords:** Total Quality Management, Hospitalization, Patient Discharge, Hospitals, Review, Gestión de Calidad Total, Hospitalización, Alta del Paciente, Hospitales, Revisión, Gestão da Qualidade Total, Hospitalização, Alta do Paciente, Hospitais, Revisão

## Abstract

**Objectives:**

to identify scientific evidence regarding the use of Lean Healthcare approach in the hospitalization and patient discharge process.

**Methods:**

this is an Integrative Review conducted in the PubMed, LILACS, SCOPUS, CINAHL, Web of Science, and Embase databases.

**Results:**

out of 904 records identified, three were included in this review. The studies demonstrated that when applied to discharge planning, the Lean philosophy brings favorable results, promoting improvements in the communication process, as well as assisting in workflow organization, with a reduction in length of stay and improvement in the quality of care.

**Final Considerations:**

although the Lean methodology presents positive results, it is considered that the application of the philosophy in healthcare institutions is still not sustainable, as it is often restricted to specific departments or services. Thus, to maximize the success of implementation, the Lean philosophy needs to be incorporated into the organizational culture, representing the greatest challenge.

## RESUMO

**Objetivos:**

identificar evidências científicas acerca da utilização do *Lean Healthcare* no processo de hospitalização e de alta do paciente.

**Métodos:**

trata-se de uma Revisão Integrativa realizada nas bases de dados PubMed, LILACS, SCOPUS, CINAHL, *Web of Science* e Embase.

**Resultados:**

dos 904 registros identificados, três foram incluídos nesta revisão. Os estudos demonstraram que, quando aplicada ao planejamento de alta, a filosofia Lean traz resultados favoráveis, promovendo melhorias no processo de comunicação, além de auxiliar na organização do fluxo de trabalho, com redução do tempo de permanência e melhoria na qualidade do cuidado.

**Considerações Finais:**

apesar da metodologia Lean apresentar resultados positivos, considera-se que sua aplicação nas instituições de saúde não é sustentável, uma vez que, na maioria das vezes, se restringe apenas a alguns setores e/ou serviços. Assim, para maximizar o sucesso da implementação, a filosofia Lean precisa ser incorporada à cultura organizacional, representando o maior desafio.

## RESUMEN

**Objetivos:**

identificar evidencia científica sobre el uso del enfoque *Lean Healthcare* en el proceso de hospitalización y alta del paciente.

**Métodos:**

se realizó una revisión integrativa en las bases de datos PubMed, LILACS, SCOPUS, CINAHL, *Web of Science* y Embase.

**Resultados:**

de los 904 registros identificados, se incluyeron tres en esta revisión. Los estudios demostraron que, cuando se aplica en la planificación del alta, la filosofía Lean produce resultados favorables al mejorar la comunicación y ayudar a organizar el flujo de trabajo, reducir el tiempo de estancia y mejorar la calidad de la atención.

**Consideraciones Finales:**

aunque la metodología Lean muestra resultados positivos, su aplicación en las instituciones de salud no es sostenible, ya que a menudo se limita a algunos departamentos o servicios. Por lo tanto, para maximizar el éxito de la implementación, la filosofía Lean debe ser incorporada a la cultura organizacional, lo que representa el mayor desafio.

## INTRODUCTION

Published in December 2013, the National Policy for Hospital Care (NPHC), within the scope of the Unified Health System (*Sistema Único de Saúde* - SUS), aims to establish guidelines for the organization of the hospital component in the Health Care Network. In this government proposal, bed management is considered a strategy to optimize bed occupancy and installed capacity, increase turnover based on technical criteria, reduce unnecessary length of stay, and create new openings for backlogged demands^(1)^.

To improve user care, this management should integrate clinical practice with the hospitalization and discharge process, preferably through the implementation of an Internal Regulation Core (NIR) or a Hospital Access and Quality Core (NAQH), with the latter being more focused on managing emergency and urgency beds^(1, 2)^.

Within the hospital, the NIR oversees the patient’s flow from arrival to discharge, encompassing activities that involve inputs and outputs in bed provision, consultations, diagnostic and therapeutic support services, and care management. It facilitates and organizes access to demands defined by the clinic, improves care and management processes, and significantly enhances healthcare^(3)^.

Upon admission, the patient undergoes registration and record-keeping before being assigned to a specific bed. This flow, from entry to actual bed occupation, involves different professionals from support services and nursing, following defined time frames based on the technical and clinical characteristics of each type of hospitalization. At the end of this flow, the patient discharge process begins, which includes the medical team’s prescription, nursing discharge planning, removal of devices, escorting the patient to the hospital exit, and subsequently organizing, cleaning, and administratively managing the transition of the bed from “occupied” to “available” status^(4)^.

Alongside bed management, indicators of hospital quality management are collected, such as length of stay, occupancy rate, turnover rate, and average patients per day^(3)^. However, despite the implementation of the strategies suggested by NPHC in 2013, bed management has proven to be inefficient in many Brazilian hospitals, with recurring situations such as delayed discharges, delayed bed turnover, and high length of stay^(5)^.

This inefficiency has largely occurred due to the challenge of finding management tools that effectively address the issues outlined in NPHC and align with institutional reality. However, despite these obstacles, a notable government initiative aimed at addressing these difficulties was the implementation of the Lean in Emergencies project^(6)^. Launched by the Ministry of Health and executed in partnership with the Syrian-Lebanese Hospital from 2018 to 2020, this project, based on the principles of Lean philosophy, aimed to reduce overcrowding in the emergency department^(6)^.

The concepts of Lean originated from Lean production or Lean Manufacturing at Toyota Motor Company in Japan between 1948 and 1975^(7)^. These concepts have been applied across various sectors, including healthcare, leading to the development of Lean Healthcare, which focuses on eliminating waste in healthcare production systems^(8)^.

At its core, Lean thinking involves the continuous elimination of unnecessary activities, encompassing clinical, support, and administrative processes. It encompasses both a set of tools and a management system^(9)^.

Moreover, the Lean philosophy promotes a method of continuous improvement that requires the commitment of all employees in the workplace, cultivating the understanding that problems are not solely due to human error but can also be attributed to flawed work processes^(8, 10, 11)^.

Given the widespread visibility, dissemination, and effectiveness of the Lean in Emergencies project at the national level, management tools based on the Lean methodology have become increasingly sought after by hospitals. These tools aim to ensure effective management of emergency department overcrowding, enhance bed turnover and availability, and reduce the average length of stay for hospitalized patients^(6, 12)^.

Furthermore, Lean production tools such as value stream mapping, kanban, poka-yoke, standardization, and continuous flow implementation, among others, along with methodologies aligned with the Lean philosophy like Lean Six Sigma, have been applied in hospital settings, particularly in the hospitalization and discharge processes. These approaches aim to improve access and care for the population^(2)^.

However, despite the growing integration of Lean thinking in healthcare services, including hospitals, there remains resistance to implementing and adopting this methodology in such settings. Objections from top management, limited knowledge of Lean philosophy and management tools within the healthcare field, reluctance among workers to embrace new challenges and activities, sporadic applications confined to specific departments rather than a collective adoption as part of the organizational culture advocated by Lean thinking, and a lack of training and preparedness for the sustainable implementation of Lean Healthcare have been reported in many cases^(3, 4, 5)^.

Given these challenges, it is crucial to explore the existing literature on the implementation of Lean Healthcare in healthcare institutions and its effectiveness in the hospitalization and discharge processes, particularly in Brazil, where scientific publications on this topic are scarce.

## OBJECTIVES

To identify the scientific evidence regarding the utilization of the Lean Healthcare approach in the hospitalization and discharge processes.

## METHODS

An integrative literature review was conducted, which involved searching, critically evaluating, and synthesizing the available scientific evidence on the topic. This method contributes to the theoretical development of the subject and explores its potential for practical application. The research process consisted of six stages: identifying the topic and selecting the research question, literature sampling or search, categorizing the selected studies, evaluating the studies included in the integrative review, interpreting the results, and synthesizing knowledge^(13)^. It is worth noting that this study followed the recommendations of the PRISMA guideline (Preferred Reporting Items for Systematic Reviews and Meta-Analyses)^(14)^.

The guiding question of the study was structured based on the PCC (Problem, Concept, and Context) strategy^(15)^. The following components were considered: P - “Hospitalization and Discharge”; C - “Lean Healthcare Methodology”; C - “Reduction of Length of Stay.” Based on these components, the following guiding question was established: “Does the application of Lean Healthcare methodology in the hospitalization and discharge processes contribute to the reduction of length of stay?”.

The search for articles was conducted in March 2022 in the following databases: National Center for Biotechnology Information (NCBI/PubMed), Excerpta Medica Database (EMBASE), Web of Science, Latin American and Caribbean Health Sciences Literature (LILACS), Cumulative Index to Nursing and Allied Health Literature (CINAHL), and SCOPUS. Controlled descriptors, such as MESH (Medical Subject Headings - MEDLINE/PubMed), DeCS (Health Sciences Descriptors -LILACS), and EMTREE (Embase), were used. Since SCOPUS and Web of Science do not have controlled vocabulary, the same descriptors were used as keywords. Chart 1 presents the search strategy outlined using terms and combinations with the use of Boolean operators.

**Chart 1:** Search strategy in selected databases

Databases	Search strategy
PubMed	*(“Total Quality Management” OR “Lean Healthcare” OR “Lean System” OR “Lean methodology”) AND (“Patient Discharge”) AND (Hospitalization)*
LILACS	*(“Total Quality Management” OR “Lean Healthcare” OR “Lean System” OR “Lean methodology”) AND (“Patient Discharge”) AND (Hospitalization)*
SCOPUS	*(“Total Quality Management” OR “Lean Healthcare” OR “Lean System” OR “Lean methodology”) AND (“Patient Discharge”) AND (Hospitalization)*
CINAHL	*(“Total Quality Management” OR “Lean Healthcare” OR “Lean System” OR “Lean methodology”) AND (“Patient Discharge”) AND (Hospitalization)*
Web of Science	*(“Total Quality Management” OR “Lean Healthcare” OR “Lean System” OR “Lean methodology”) AND (“Patient Discharge”) AND (Hospitalization)*
Embase	*(“Total Quality Management” OR “Lean Healthcare” OR “Lean System” OR “Lean methodology”) AND (“Patient Discharge”) AND (Hospitalization)*

The search for full-text studies was conducted through the Coordination for the Improvement of Higher Education Personnel journal portal. The articles were exported to the Rayyan QCR platform, which was used to manage the identified studies. This platform assisted in identifying and removing duplicate articles, as well as determining eligibility criteria based on titles and abstracts^(16)^. It should be noted that two independent reviewers conducted the study, using blinding tools, and a third reviewer was consulted in case of discrepancies. However, it is worth mentioning that there was agreement between the two reviewers regarding the inclusion of potential studies in the sample, and the involvement of a third evaluator was not necessary.

The following inclusion criteria were defined: primary articles available in full text, published in English, Spanish, or Portuguese between 2011 and 2021, addressing the study topic. Exclusion criteria included studies that solely used the “Six Sigma” methodology or combined “Lean Six Sigma,” case studies, literature reviews, and editorials.

For the extraction and categorization of the included studies, the instrument developed and validated by Ursi and Galvão^(17)^ was used. This instrument is divided into article identification, methodological characteristics of the study, and key findings.

The level of evidence was also determined according to the proposal by Melnyk and Fineout-Overholt^(18)^, which classifies it as follows: I - Systematic review or meta-analysis; II - Randomized controlled trial; III - Non-randomized controlled trial; IV - Case-control or cohort study; V - Systematic review of qualitative or descriptive studies; VI - Qualitative or descriptive study; VII - Expert opinion or consensus.

After extracting the results, the data were organized and presented according to thematic categorization.

## RESULTS

**Figure 1:**
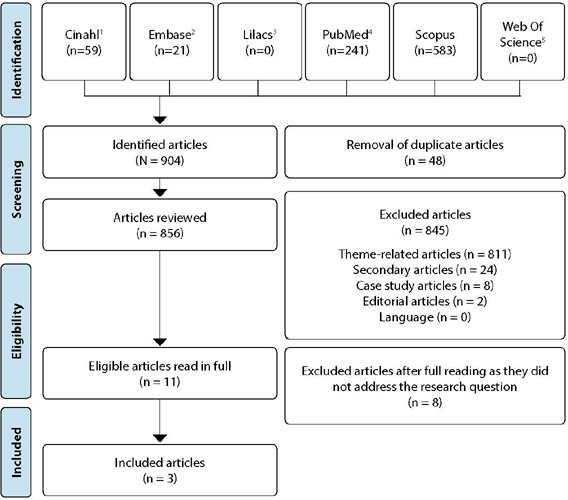
PRISMA Flowchart of Article Selection Process for the Integrative Review^(14)^

A total of 904 records were identified in 4 out of the 6 selected databases, namely PubMed, SCOPUS, CINAHL, and Embase. No studies were found in the LILACS and Web of Science databases. Among all the articles, 48 duplicates were excluded from the databases. Out of the remaining 856 articles, 845 were excluded after reviewing their titles and abstracts as they did not meet the inclusion criteria. Subsequently, the full texts of the remaining 11 articles were read, and three of them were included in the present review (Figure 1).

*^1^Cumulative Index to Nursing and Allied Health Literature (CINAHL); ^2^Excerpta Medical Database (EMBASE); ^3^Latin American and Caribbean Health Sciences Literature (LILACS); ^4^National Center for Biotechnology Information (NCBI/PubMed); ^5^Web of Science.*

It should be noted that the three studies were conducted in North America, specifically in the United States. Overall, there is variability in the sample sizes and participant characteristics, highlighting the heterogeneity of the explored contexts.

Regarding the publication years, one study was published in 2014 (n=1), another in 2016 (n=1), and the third in 2018 (n=1). It is worth mentioning that the studies were conducted in different hospital institutions, units, and departments, demonstrating the diversity of explored contexts.

Furthermore, regarding the journals in which the selected articles were published, it was found that one article was published in a medical sciences journal, one in a journal related to trauma and acute care, and one in a journal focused on emergency medicine. As for the methodological design and level of evidence, all three studies were classified as level III evidence, using a quasi-experimental design (before and after). In this type of study, there is no prior randomization to allocate participants into control and intervention groups, as participant selection is at the discretion of the researcher. Therefore, in this design, it is not possible to control for other factors that may occur simultaneously with the intervention. It is worth noting that although this review did not employ bias analysis tools for intervention studies, the three studies showed a certain rigor in defining the inclusion and exclusion criteria for the sample^(19)^.

The objectives of the articles found were: (1) to analyze whether the new model of multidisciplinary visits based on Lean is associated with a decrease in patient length of stay, an increase in discharges before noon, and the establishment of an estimated discharge date and patient satisfaction^(20)^; (2) to reduce hospitalization time and improve the quality of care through a multidisciplinary team approach^(21)^; (3) to improve communication and discharge planning through process mapping^(22)^. The favorable results obtained included: increased discharges before noon, increased documentation of estimated discharge date, reduction in patient length of stay, reduction in team rework, and improvements in institutional processes^(20,21,22)^.

The Chart 2 presents a summary of the articles included in the integrative review, according to title, year/country, design and number of participants, interventions and outcomes.

**Chart 2:** Summary Chart of the Articles Included in the Integrative Review

Title	Year/Country	Design and Number of Participants	Interventions	Outcomes
Lean-Based Redesign of Multidisciplinary Rounds on General Medicine Service^(20)^	United States, 2018.	Quantitative approach; Design: quasi-experimental (pre- and post-intervention). n = 6,085 patients.	Hospital Digital System, using the “Press Ganey Survey” instrument.	Discharges before noon increased from 6.9% to 10.7% (p<0.001). The recording of estimated discharge dates increased from 31.4% to 41.3% (p<0.001). However, there was no change in patient satisfaction.
Lean methodology for performance improvement in the trauma discharge process^(21)^	United States, 2014.	Quantitative approach; Design: quasi-experimental (pre- and post-intervention). n = 4,000 admissions annually.	Satisfaction Survey.	Changes focused on a standardized morning report structure resulted in a decrease in the number of unanswered consultation questions from 67% to 34% (p=0.0021). Physical therapy rework was reduced from 35% to 19% (p=0.016). Patients admitted to non-trauma service units had 1.6 times longer length of stay (p<0.0001).
Use of a Dedicated, Non-Physician-led Mental Health Team to Reduce Pediatric Emergency Department Lengths of Stay^(22)^	United States, 2016.	Quantitative approach; Design: quasi-experimental (pre- and post-intervention). n = 1,640 visits.	Electronic Medical Record and Emergency Services Security Database.	There was a statistically significant decrease in the mean emergency department length of stay (332 minutes vs. 244 minutes, p<0.001). Significant reductions were also observed in the median emergency department length of stay (225 minutes vs. 204 minutes, p=0.001), physical security interventions (2.0% vs. 0.4%, p=0.004), and use of restraints (1.7% vs. 0.1%, p<0.001).

## DISCUSSION

To analyze the publications included in this study and facilitate discussions, the selected articles were categorized into three groups: discharge planning, length of stay, and care outcomes. It should be noted that the same article may be included in multiple categories.

### Discharge Planning

Discharge planning for hospitalized patients begins early in their stay and aims to provide individualized, patient-centered, and compassionate care while reducing the length of stay and preventing complications that may lead to readmissions.

Within the discharge planning category, two studies were included that focused on implementing Lean methodology to improve the discharge process. Both articles identified inefficient communication among team members as a major barrier. The studies utilized Lean tools such as process mapping, value stream management, rounds, workshops, and multi-disciplinary visits^(20, 21)^.

Lack of communication and planning among the interdisciplinary team contribute to delayed discharge, resulting in longer hospital stays. It is crucial to identify the reasons for these delays, as it is the first step toward finding solutions and implementing changes in work processes to improve outcomes^(23)^.

Process mapping and value stream management are valuable Lean tools for identifying inefficiencies and waste in the discharge process and addressing them based on the hospital’s specific context^(20, 21)^. Implementing process mapping helps reduce errors, enhance care delivery efficiency through standardized procedures, and reorganize departments, ultimately benefiting patients. Additionally, it fosters integration and improved communication among staff members^(24)^.

Multidisciplinary rounds serve as an intervention strategy to enhance communication and discharge planning. These rounds involve structured daily communication among key members of the care team, ensuring that everyone is updated on the patient’s care plan and prepared for discharge^(20)^.

Multidisciplinary teamwork plays a crucial role in the discharge planning process, as patient recovery is influenced by various factors. In addition to medical treatment and nursing care, patients rely on services such as physiotherapy, psychology, social work, nutrition, and others. Effective communication among team members is vital in this context.

The round is another valuable Lean tool that promotes communication. It involves brief daily meetings with all team members to address urgent issues, improve communication, and streamline the discharge process. Quick process improvement workshops and active daily management are additional Lean strategies employed to enhance communication^(20, 21)^.

Therefore, it is clear that the Lean methodology can be applied from the early stages of the discharge planning process, including identifying root causes and using appropriate tools to address them. Communication is a critical aspect of healthcare processes, and its inefficiency is recognized as a significant problem. Thus, working on improving communication through management tools is essential for effective clinical and managerial processes. The studies demonstrated that when the Lean methodology is applied to discharge planning, it leads to favorable outcomes and improvements in communication processes^(20, 21)^.

### Length of Stay

Two studies were included in this category as they focused on the length of stay for hospitalized patients and the application of Lean methodology to improve this process^(20, 22)^. The length of hospital stay is of utmost importance for healthcare institutions as prolonged stays can increase patients’ susceptibility to infections, antibiotic resistance, and mortality^(25)^.

Machado and Machado^(23)^ observed that in 50% of the studies, the impact on bed management was attributed to increased length of stay. Additionally, 21% of the studies reported increased occupancy rates, overcrowding, and waiting lists for admission.

Factors associated with prolonged length of stay include the use of invasive devices, sepsis, extended stays in the emergency department, prolonged use of invasive devices, and prolonged antibiotic use. Patients affected by healthcare-associated infections also experience longer stays^(26)^.

A cohort study conducted in a tertiary care hospital in Singapore identified the development of methicillin-resistant Staphylococcus aureus in patients hospitalized for 7 to 13 days. The proportion of cases was 2.7 times higher than in patients hospitalized for 1 to 6 days and increased to over 50 times higher in patients hospitalized for more than 63 days^(27)^.

Identifying factors associated with prolonged length of stay can help healthcare managers identify areas of improvement and develop plans that optimize resources and achieve excellence in healthcare delivery^(26)^.

Therefore, it is important for healthcare institutions to have knowledge of patients’ nosological profile in order to develop management tools that meet their needs and the needs of their team, resulting in satisfactory outcomes for all.

A study by Kane et al.^(20)^ demonstrated an increase in discharges before noon, reflecting improved turnover and optimization of inpatient beds. The study by Uspal et al.^(22)^ showed a reduction in the length of stay for patients in a pediatric psychiatric emergency department. Initially, process mapping was used to identify waste, and adjustments were made to the physical environment, visitor guidance, and medical care organization. These results align with a Brazilian study that found a significant increase in hospital discharges and improvements in utilization and turnover indicators through Lean methodology implemented by the NIR^(3)^.

The study by Uspal et al.^(22)^ also emphasized the importance of both external and internal customer voice in the pursuit of a new care model. Lean methodology aims to identify what is valuable to the external customer to maximize their satisfaction. On the other hand, the internal customer highlights problems resulting from process failures and contributes to achieving the best healthcare outcomes.

### Healthcare Outcomes

In the category of Healthcare Outcomes, the included articles presented outcomes after implementing Lean methodology. Three studies were included in this category^(20, 21, 22)^, and two of them demonstrated improvements in healthcare outcomes following process modifications.

Patient-centered care is recognized as a fundamental value in multidisciplinary healthcare. It is associated with positive outcomes, such as reduced complaints of neglect, improved satisfaction among the healthcare team due to patient delight experiences, increased treatment adherence, waste reduction, and cost savings^(28)^.

Two of the reviewed articles reported favorable results related to the work of the multidisciplinary team in patient care after implementing Lean tools. In the study by Uspal et al.^(22)^, an improvement was observed in the care of pediatric psychiatric patients in a pediatric hospital, with a reduction in physical restraint and physical security interventions after team training. There was also an improvement in internal and external customer satisfaction, a reduction in potential adverse events caused by prolonged patient stays in the emergency area, and a decrease in the time to initiate therapy for inpatients.

The study by O’Mara et al.^(21)^ demonstrated a reduction in physiotherapy rework from 35% to 19% over an 8-month period. Healthcare outcomes are of utmost importance in studies as they make a difference in the satisfaction of both the healthcare team and the patients.

A survey conducted in a university hospital in Rio Grande do Sul, Brazil, with nursing workers in an emergency department identified factors of dissatisfaction, such as workload overload, overcrowding, frustration in being unable to meet patient needs, lack of problem resolution by management, high workload demands in the hospital environment, lack of material and physical resources, and stretchers in the corridors hindering the flow of people^(29)^.

The studies by O’Mara et al.^(21)^ and Uspal et al.^(22)^ demonstrated that the use of Lean methodology resulted in increased patient satisfaction. In the study by O’Mara et al.^(21)^, the improvement in satisfaction stems from the enhancements made in the discharge instructions provided to patients. In the study by Uspal et al.^(22)^, the implementation of Lean redesigned the care in the emergency department, leading to higher patient satisfaction compared to the previous period.

However, this factor needs to be identified and addressed by healthcare institutions as it also reflects on patient healthcare outcomes. Researchers point out that the application of Lean in healthcare institutions contributes to the emergence of patient-related outcomes, such as user satisfaction and patient safety^(9)^.

### Study limitations

This study highlighted the need for investment in research focused on Lean principles in a national context, as all studies included in this review were conducted in an international setting. Furthermore, there is a need to invest in more consistent, rigorous, and robust methods that can be applied in practice, assisting in the organization of hospitalization and discharge processes. Additionally, future studies may provide a more in-depth analysis of patient satisfaction following the implementation of the methodology, the use of care indicators to achieve patient-centered outcomes, sustainability indicators for improvement processes, and cost analysis.

### Contributions to the healthcare field

This study revealed that implementing the Lean methodology in the hospitalization and discharge process has the potential to significantly reduce patients’ length of stay in the hospital, with a potential impact on reducing costs associated with hospitalization.

## FINAL CONSIDERATIONS

Considering the scarcity of scientific studies on this topic, especially in Brazil, this study did not gather a large quantity of articles. However, the included works demonstrated the relevance of using Lean management tools in the hospitalization and discharge processes, which resulted in a reduction in length of stay, positively impacting communication, optimizing bed utilization, and ultimately improving patient satisfaction. Despite the Lean methodology contributing to achieving positive and robust results, it is evident that most healthcare institutions do not ensure the sustainability of applying this philosophy. To achieve successful implementation, the Lean philosophy needs to be incorporated into the organizational culture, which represents a more complex reality. Lastly, the importance of conducting long-term studies to assess the real impact of implementing this philosophy is emphasized, as it is the true key to continuous improvement.
